# Cultural evolution from the producers’ standpoint

**DOI:** 10.1017/ehs.2023.20

**Published:** 2023-08-09

**Authors:** Jean-Baptiste André, Nicolas Baumard, Pascal Boyer

**Affiliations:** 1 Département d'Etudes Cognitives, Ecole Normale Supérieure, Paris, France; 2 Department of Psychology, Washington University in St Louis, St Louis, MO, USA

**Keywords:** cultural evolution, fitness costs, social evolution theory, evolutionary psychology, symbolic culture

## Abstract

Standard approaches to cultural evolution focus on the recipients or consumers. This does not take into account the fitness costs incurred in producing the behaviours or artefacts that become cultural, i.e. widespread in a social group. We argue that cultural evolution models should focus on these fitness costs and benefits of cultural production, particularly in the domain of ‘symbolic’ culture. In this approach, cultural products can be considered as a part of the extended phenotype of producers, which can affect the fitness of recipients in a positive way (through cooperation) but also in a detrimental way (through manipulation and exploitation). Taking the producers’ perspective may help explain the specific features of many kinds of cultural products.

## Graphical abstract

**Figure:**
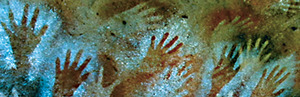

**Social media summary:** Explaining culture: why we need to consider the fitness interests of producers of cultural material.

Evolutionary models of human culture must address two fundamental questions: why do humans create cultural materials *at all*; and why do humans create *these particular forms* of cultural materials? We contend that some standard approaches to cultural evolution are insufficient to address these two questions, because they mostly focus on *consumption* or *reception* and tend to neglect the *production* side of cultural phenomena.

That is particularly clear in what we will call, for lack of a better term, the domain of ‘symbolic culture’, that includes art, narratives, religious representations, games, sports, ethnic ideologies, religious representations and superstitions, moral norms and codes and many social conventions. This obviously is a disparate domain. One common feature is that these different cultural phenomena seem difficult to explain in terms of fitness advantages. Why do humans compose narratives? Why spread and transmit representations of spirits and gods? How do we connect the recurrent forms of such productions in many cultures to evolved capacities and preferences?

In this ‘symbolic’ domain, a common answer to such questions is that these cultural productions are by-products of our evolved mental architecture. They invade minds as viruses and parasites invade bodies (Dawkins, 1976: 189–201) or they constitute super-stimuli, ‘cheesecake for the mind’ (Pinker, 1997: 524ff.) (see discussion in Box A). Those are insufficient explanations if we want to understand why cultural objects of this kind are produced at all. From an adaptationist perspective, some agents must have a fitness advantage in creating symbolic culture, as there is no such thing as free cheesecake.

Box A. Models of cultural evolution: the consumers’ side

The study of cultural evolution has known a spectacular development in the last 20 years, as evolutionary anthropologists applied formal quantitative models to the available evidence (Gray, Bryant, & Greenhill, 2010; Shennan, 2011), for instance phylogenetic techniques in the study of language families (Atkinson, 2011; Gray et al., 2010). What explains the particular trends described by such models? Proposals are diverse in this matter and the field is subject to substantial theoretical debates.

These proposals differ in terms of the psychological adaptations they describe as underpinning cultural evolution. At one end of the spectrum, dual inheritance theory (Boyd & Richerson, 1985) considers that individuals acquire social information on the basis of minimal heuristics, mostly akin to some form of imitation, and that the selection of materials to imitate is mostly sensitive to properties of the *sources* of information and its diffusion, and less so to the *content* of the information transmitted. For example, these theories attach great importance to simple heuristics such as conformist bias (imitation biased by the relative frequency of a cultural item) or prestige bias (imitation biased by the general social prestige of its bearer) (Henrich & Gil-White, 2001).

At the other end of that spectrum, cultural attraction theory or epidemiology of culture (Claidière, Scott-Phillips, & Sperber, 2014; Sperber & Claidière, 2006) considers that the acquisition and reconstruction of cultural information in individual minds is governed by numerous domain-specific capacities and preferences, such as, e.g. intuitive psychology, intuitive biology, coalitional psychology, moral intuitions, etc. – see also Tooby and Cosmides (1992). The framework emphasises the key role of the individuals’ evolved abilities to modify, compare and combine different sources of information, in creating recurrent representations in a community.

Many other models of cultural evolution are positioned between these extremes on the spectrum. For example, models proposed by Lehmann, Feldman and Foster (2008) consider relatively crude and content-free heuristics of social learning, whereas Enquist, Eriksson and Ghirlanda (2007) consider finer-grained, content-biased learning mechanisms. In other models, a specific description of the psychology of learners is required for particular domains. Language evolution, for instance, can be described as resulting from iterated learning, in which Bayesian agents use the available evidence to adjust the probability of grammars (Griffiths, Kalish, & Lewandowsky, 2008). These models show that the cultural evolution of language towards increasingly learnable grammars is highly dependent on the learners’ psychological priors (Smith & Kirby, 2008), which is confirmed by studies of artificial cultural transmission in the laboratory (Kirby, Cornish, & Smith, 2008).

A common feature in all these models, beyond the differences, is the common assumption that we should first and foremost consider characteristics of the receivers or consumers of cultural information, in order to explain patterns of cultural evolution.

## 1. Culture from communication

The term ‘cultural’ designates traits or behaviours with a certain amount of within-group similarity and between-group differences. In this very broad sense, a great number of factors can result in group differences, from climate to demography to sex-ratios to, most importantly, ecological conditions (Micheletti, Brandl, & Mace, 2022). Here, we only consider the sub-domain of group-specific behaviours or representations that result from human interaction and communication, using the term ‘cultural’ in the stricter sense more common in current cultural anthropology. Specifically, we call ‘cultural’ those mental representations that happen to be common among the members of some group, as a result of communication (Boyd & Richerson, 1996, 2005; Sperber, 1996).

The fact that human communication is involved has direct consequences for the study of cultural evolution. To explain the occurrence and the recurrent forms of cultural productions, one must consider what cognitive processes turn private representations (e.g. a memory of a narrative) into public representations (a verbal telling of the story) and vice versa (Sperber, 1996, 2006), in such a way that some but not all mental representations become widespread in a group. That is why models of cultural evolution generally include some description of the mental ‘biases’ or evolved predispositions that influence the acquisition and transmission of representations (see Box B for details).

Box B. Cultural ‘viruses’ and ‘cheesecake’: the limits of analogies

Why do people spend time listening to music or stories? Why do they believe in supernatural agents, magic or traditional medicine? In some domains, one may argue that the consumer benefits. For instance, fiction may be understood as training for social interaction (Gottschall, 2012; Mar & Oatley, 2008). Visual aesthetics may train perceptual systems to relevant aspects of the natural world (Tooby & Cosmides, 2001). Yet in many other domains of culture, that is simply not the case. It seems difficult to explain the cultural spread of e.g. religion, horror movies or pornography, in terms of consumer fitness.

That is why such cultural items are often described as mental viruses and ‘memes’ (Dawkins, 1976: 189–201) or ‘cheesecake for the mind’ (Pinker, 1997: 524ff.). For instance, because humans are motivated by parental investment, their nurturing instincts can be parasitised by kittens and puppies (Archer, 2011); because humans are socially vigilant, they would be attracted to the notion of witches described in the same terms as predators (Boyer, 2000).

The use of terms like ‘virus’ or ‘parasite’ illustrates what is missing here. In biological evolution, we must study the fitness of *both* host and parasite to understand their interaction, construed as an evolutionary conflict between sophisticated immune systems, on one side, and sophisticated mechanisms to escape those systems, on the other. So, if we consider some cultural information as informational parasites, we should address the question: who benefits? Whose brains have been shaped by natural selection to be willing and able to produce information perfectly suited to get into people's minds?

Just as there always remain biological parasites capable of bypassing their hosts’ immune systems, there always remains a certain amount of harmful cultural information that consumers are not able to filter out. So cultural ‘parasitism’ occurs when some individuals invest brain power, effort and resources to find the right way to divert another brain's attention, e.g. from fitness-relevant stimuli like human speech towards artificial stimuli like musical sounds. So-called cultural viruses are the products of individual minds trying to achieve effects on the minds of others (getting others’ attention, signalling some qualities, manipulating others, sending some information, etc.) for their own benefit.

In this sense the cheesecake metaphor is partly appropriate, as it suggests that producers of cultural objects, just like pastry chefs, need to be attuned to the evolved preferences of consumers. The metaphor becomes misleading, however, in that the nutritional benefits of super-stimuli like cheesecake are not needed in the modern world, and come with detrimental side-effects. To the contrary, we argue that in many cases cultural production occurs and is sustained, because it confers actual advantages to consumers as well as producers – more akin to healthy food than to unnecessary sweets.

In the domain of ‘symbolic’ culture, this psychological strategy has been very productive. For instance, the fact that musical traditions, however diverse, abide by similar tonal principles (Huron, 2006; Thomson, 1958) is explained by properties of the human auditory cortex and memory; the most successful stories follow a limited set of narrative formats (Boyd, 2010; Gottschall, 2012), because they satisfy a human motivation to know and understand the social lives of others; people construe ethnicity in terms of innate properties of individuals, because of our intuitive essentialism about natural categories (Gil-White, 2001), and so forth.

Note that these are all consumer-centred models. The exclusive focus on consumption, unfortunately, does not provide an explanation for the large investment in the *production* of those behaviours or artefacts, and how that investment may influence their form. For instance, listening to Mozart or the Beatles is indeed riveting (on the consumption side), but that does not by itself explain the thousands of hours of training invested by composers and performers. In many domains of cultural knowledge, production is less dramatically costly but still begs for an explanation. Why do people expend cognitive resources creating complex stories, recipes for dealing with misfortune and ideas about gods and spirits?

To make this question more precise, we must keep in mind that the production of potentially ‘cultural’ representations consists in behaviours that affect the distribution of mental representations among a group of receivers. For instance, telling a new story creates new memories in those who heard it. Telling people about the danger from immorality may affect the spread of specific moral emotions and motivations in the audience. So models of cultural evolution would consider both a proximate question – what human capacities and motivations are involved in trying to modify other agents’ representations via cultural productions – and an ultimate question – what selective pressures would account for these specific capacities and motivations? To reprise Tinbergen's famous four questions (Tinbergen, 1963), we do not address questions of ontogeny or phylogeny here, but only consider mechanism and function.

## 2. Production is not consumption: different mechanisms, different interests

Production and consumption differ. First, obviously, they most often consist of different behaviours that engage different sets of capacities and preferences – compare musical performance and musical enjoyment, story-telling skills and attention to narratives.

More important, production and consumption generally engage different interests. The pleasure that an audience derives from listening to Mozart or the Beatles does not in itself constitute a benefit to the composers and performers. Conversely, the benefits that shamans, healers or priests may derive from their knowledge are not directly aligned with the benefits x2013; or costs, as a matter of fact – for their customers. As a result, the fact that a cultural item is useful or not for its consumers does not in and of itself explain its success, or lack thereof. So, to explain the existence and nature of symbolic culture, one must explain why producers spend time and energy producing them in the first place. Put differently, an adaptationist approach to psychology predicts that producers should have a motivation to generate or modify cultural items if and only if this very activity will eventually have a positive impact on their inclusive biological fitness. That is, the eventual success of a given cultural item, that is, the *cultural* fitness of that item, is conditioned by the effect of producing that item on the *biological* fitness of its producer (see also El Mouden, André, Morin, & Nettle, 2014; Micheletti, 2020; Nettle, 2020). This inclusive benefit to the producer may be accompanied, depending on the situation, by a benefit or a cost to the inclusive fitness of the consumer.

A convenient framework to describe and understand this logic is social evolution theory (Hamilton, 1964). The production of cultural information is a social behaviour, whereby an individual, the producer, acts upon another individual, the consumer. Cultural production can therefore be classified into four categories according to its effects on the reproductive success of both the producer and the consumer (Hamilton, 1964; West, Griffin, Gardner, & Diggle, 2006; see Table 1).

**Table 1.** Types of social behaviour, after Hamilton (1964)

		Effect on recipient	
		Positive	Negative
Effect on actor	Positive	Mutualism	Selfishness
	Negative	Altruism	Spite

In what follows, we consider these different types of social effects and show how, in each case, the interests of producers and consumers interact to explain the features of symbolic culture. Our aim here is mostly illustrative, as we cannot provide a detailed explanation of the occurrence of these types of interaction in all domains of culture.

## 3. Diverse configurations of interests

### 3.1 Altruistic production with indirect benefits

The production of symbolic culture is altruistic if it has a negative effect on the direct fitness of the producer and a positive effect on the direct fitness of the consumer (Hamilton, 1963). The producer can ultimately benefit only if the consumer's benefit constitutes an indirect fitness benefit, that is, if the consumer is a genetic relative. Social evolution theory predicts that altruistic cultural production will be mostly directed at close kin, as a function of perceived relatedness.

#### Cultural examples

Parents the world over engage in pedagogic interaction, whereby infants spontaneously attend to generic information, when caretakers attract their attention with specific communicative cues (Csibra, 2007). Acquiring general knowledge of the world is beneficial to infants and in turn indirectly to their genetic relatives. In a similar way, infant-directed singing is universal (Mehr, Singh, York, Glowacki, & Krasnow, 2018). The infant benefits from sleep and reassurance, and it is also in the parent's genetic interest to promote their offspring's good health.

### 3.2 Selfish production: conflict and manipulation

The production of cultural information is termed selfish if it has a positive effect on the fitness of the producer and a negative effect on the fitness of the consumer. The producer has a direct interest in producing cultural information but this production generates costs for the consumers. The interests of producers and consumers are not aligned, so that there is an evolutionary conflict between them. In this case, to obtain her benefit, the producer must *manipulate* the consumer, i.e. she must use the fact that the consumer does not have a perfect ability to filter information.

#### Cultural examples

We can sometimes identify aspects of symbolic culture as mostly manipulative. That is for instance the case for those religious cults whose leaders extract resources (labour, money, sexual services, etc.) from group members (Dawson, 1998). Yet the producer/consumer asymmetry also extends to less dramatic cases. Consider for example religious activities in small-scale societies. Notions of spirits and ancestors are attention-grabbing, which explains people's interest (Boyer, 1994). That much accounts for the consumption side. Yet we also know that in all human societies there are specialists such as diviners, shamans, etc. who produce most of the current religious representations and offer religious goods and services, in the form of rituals, amulets, incantations, etc. (Boyer, 2019; Singh, 2018; Winkelman, 1990). The activities promoted by these specialists do not generally bring much benefit to the consumers, and may indeed be costly in some cases. For producers in contrast, they are the source of social status, material resources and prestige that may translate into direct fitness benefits. This amounts to a manipulative interaction, to the extent that there is no advantage for the consumers (this of course is an empirical question, and the answer is case specific).

### 3.3 Mutually beneficial production

The production of cultural information is mutualistic if it has a positive effect on the direct fitness of both producer and consumer (Hamilton, 1964; West, Griffin, & Gardner, 2007). Just like mutualistic cooperation in general, mutualistic cultural production can take place for two distinct reasons: (a) it can benefit both the consumer and the producer in an automatic manner; and (b) the producer can derive a benefit contingently, through a positive response of the consumer, a mechanism called reciprocity (in a broad sense of the term), conditional cooperation or sometimes ‘enforcement’ (West et al., 2007). We consider these two possibilities in turn.

#### (a) Mutualistic cooperation with automatic benefits

Producing a cultural item that is useful to a consumer automatically benefits the producer if they have a common interest. The alignment of producer and consumer interests plays a significant role in culture in cases where the behaviour produced is used by the consumer as an honest signal about the quality of the producer. That is the case in sexual selection for instance. The preferences of the consumer (e.g. females) create a selection pressure for specific traits, capacities and behaviours in the male producer (Darwin, 1871; West-Eberhard, 1979), and both sides benefit as long as the signal is honest rather than manipulative.

##### Cultural examples

Signalling of this kind may provide a motivation for the production of cultural objects. A good illustration is the invention of sport, i.e. of rule-bound public displays of physical qualities, found in the most diverse cultural environments, with a clear gender imbalance in most non-modern cultures (Wiedemann, Barton, & Hill, 2012). Sportive activities generally advertise the physical qualities of individual males or coalitions of men, including heritable qualities like coordination, explosive strength and dominance (De Block & Dewitte, 2009: 4). The fact that precise and constraining rules govern these behaviours turns possibly multi-dimensional differences between individuals into clear rankings that provide proxies for mate value and thereby may affect reproductive fitness (Miller, 1999: 253). In that sense, sports provide a functional equivalent of courtship displays. They may also serve males by providing them with a clear indication of each other's relative formidability. With similar goals, ritual ceremonies may include sport-like displays. Consider for instance the famous Melanesian land dives, the (highly dangerous) ancestor of bungee-jumping, used in Pentecost and other islands as a demonstration of male warrior-like qualities towards both women and men (Jolly, 1994).

#### (b) Mutualistic interaction with conditional cooperation

In most cases, producers do not automatically benefit from their production but eventually gain nevertheless through an exchange with consumers. The possibility of cooperation based on conditional exchanges, in humans, dramatically expands the range of situations in which individuals can benefit from producing new pieces of information, because it allows the value of cultural information to consumers to eventually spill over to producers.

##### Cultural examples

There may be mutual advantages for both producer and consumer, in many cultural domains usually described only from the consumer's side. Consider ‘traditional’ pre- or para-scientific forms of medicine. They do include all manners of effective cures, based e.g. on empirical knowledge of plants or on fracture-reduction techniques, see (McDade et al., 2007) for example. These traditions also include a large number of activities with no positive (and often some detrimental) effects on physiology, as in the widespread practice of bloodletting. The point also applies to early Western medicine, which until the beginning of the twentieth century had very limited therapeutic efficacy (Wootton, 2007). In cultural evolution models, this has been mostly explained from the consumers’ standpoint. For instance, bloodletting was congruent with universal intuitions to the effect that pathologies result from foreign vectors invading the body (Miton, Claidière, & Mercier, 2015). Yet we should also consider that there are benefits for producers, as specialists in traditional medicine accumulate reputational advantages (with potential fitness gains) from the patients’ belief in their efficacy. In fitness terms, one could describe this interaction as (sometimes) mutually beneficial. That occurs when producers gain reputation while consumers gain well-being via placebo responses to medication, observed in both modern and traditional medical contexts (Price, Finniss, & Benedetti, 2008). (Note that in this case the consumers’ benefits may be real although they are not the benefits promised by providers.)

The production and transmission of conventional norms also illustrate these mutualistic advantages, in domains as diverse as market interactions, children's games, highway regulations and dress codes. Usually, we consider that conventional norms provide advantages to consumers. Given that large domains of social interaction include (or consist in) coordination games, the existence of arbitrary coordination points (e.g. that a handshake is done with the right hand) is to the consumers’ advantage – a point emphasised in conventional accounts of norms (Bicchieri, 2006: 11–28; Lewis, 1969). Yet that is also why there is an advantage in providing such norms when they are absent, and (more relevant to actual social interaction) to maintaining norms against possible deviations, either instigated by interested parties, or simply as the result of entropy in communication. Surprisingly, this logic may also apply to constraining or coercive norms, as there may be advantages in both imposing the rules and abiding by them (see discussion in Box C).

Box C. Could constraining norms be an example of cooperation?

Norms are often described as external to individuals, as sets of rules imposed on them. Yet norms are created and (more often) re-formulated and upheld by particular individuals who may derive benefits from the widespread adoption of the norm.

Consider for instance norms related to common pool resources, such as pastures, fisheries, canals, etc. In many societies, very specific norms regulate access to these resources, and violators are punished either formally or informally (Ostrom, 2005). From a consumer's standpoint of view, these rules may seem coercive. However, such norms may constitute a standard example of mutualistic interaction with conditional cooperation. Individuals contribute to the enforcement of the norm (through time or resources given to the institution) in exchange for a higher level of cooperation from their partners. Here, norms instantiate second-order cooperation, i.e. a cooperative interaction that makes another (first-order) cooperative interaction more efficient. For instance, the members of Indonesian rotating credit associations produce and enforce norms regarding their meeting (e.g. weekly meetings are mandatory, they always take place at the same time, at the same place) because regular meetings facilitate the monitoring of the members of the association (Fessler, 2002). In line with reciprocity theory, the most stable cultural norms regulating common pool resources are based on considerations of proportionality and fairness (Baumard, 2015).

The same point can be made for norms regulating sexuality. From the customers’ viewpoint, puritanical norms may be, in appearance, essentially coercive to individuals. Why would they adopt a restrained lifestyle, depriving themselves of easy sex and drugs? However, if we take the producers’ standpoint into account, puritanical norms may be in fact, under certain circumstances, mutually advantageous. They increase the cost of sexual promiscuity, making it more advantageous to engage in stable pair-bonding and parental investment, for both men (reduced risk of cuckoldry) and women (reduced risk of desertion). So it becomes advantageous for individuals in such a situation to produce and enforce norms regulating pornography, alcohol consumption, abortion, pre-marital sex or masturbation (Fitouchi, Baumard, & André, forthcoming; Kurzban, 2012: 72ff.; Kurzban, Dukes, & Weeden, 2010). Consistent with this interpretation, puritanical or restrained norms are more likely to occur in societies where stable pair-bonding and high parental investment are likely to be advantageous strategies (Baumard & Chevallier, 2015; Baumard, Hyafil, Morris, & Boyer, 2015).

This explanation may also apply to the more extreme case of patriarchal norms that regulate the place of females in the society, requiring them to behave very modestly, prohibiting certain behaviour, preventing them to be educated and to increase their welfare, in the name of vaguely defined principles like ‘honour’, ‘modesty’ or ‘purity’ (Afkhami, 1995). Although these norms seem to organise the manipulation of female behaviour by powerful males, anthropologists report significant support for such norms, including by some women (Abu-Lughod, 1986). Consistent with the mutualistic pathway described here, it may be the case that patriarchal rules, like puritanical norms, confer (distinct) advantages to (some) men and women as they raise the cost of promiscuity.

#### (c) Consequences of mutualism

Note that it is not always a simple (or necessarily productive) endeavour to try to evaluate the extent to which cultural production belongs to either one of these forms of mutualism. For instance, norms may in some cases result from the producer's interest, and confer benefits on consumers as a by-product, while in other cases they directly benefit both. What is more important for explaining cultural evolution is that in both cases, the fitness interests of producers and consumers are lined up.

### 3.4. Spiteful production

For the sake of completeness, we must mention the situation in which the producer pays a cost in terms of *direct* fitness in order to produce cultural information that manipulates the consumer to the detriment of his or her fitness. Such spiteful behaviours can only be favoured by natural selection if they have a positive effect on the producer's indirect fitness (Lehmann, Bargum, & Reuter, 2006; West & Gardner, 2010), which can typically occur when the recipient is competing with close relatives of the producer, so that the negative effect on the recipient indirectly has a positive effect on these relatives. This situation is probably rare, however, and does not play a structuring role in cultural evolution.

### 3.5. Different fitness paths in the same domain of cultural products

Note that these pathways can be combined. Above, we mentioned some typical examples for each configuration of interests, for the sake of illustration. Yet a domain of cultural production may be favoured by distinct fitness dynamics in different situations. There may be a large element of sexual selection in the production of modern music, art and narratives (Miller, 1999), although that interpretation is contested for Palaeolithic art as creators were also female (Snow, 2006). In some cases musical performers extract resources from consumers, so that the manipulation dynamic is the relevant one. There may also be situations in which there are actual advantages for the consumers of art and fiction, so that the dynamic is a cooperative one. Traditional medicine may be considered as mostly exploitative (in the evolutionary, not psychological sense), but inasmuch as it delivers advantages it could be sustained in cultural evolution by a cooperative equilibrium. Which of these evolutionary dynamics is relevant, for each cultural product, is of course an empirical matter.

## 4. Producers’ interests explain properties of symbolic culture

In our view, seeing cultural items in terms of producers’ interests may generate substantive hypotheses about the observed general features of some cultural products. As stated above, the goal of cultural evolution models is to explain not just why there is culture (mental representations recurrent as a result of communication) but also why some particular features are recurrent. Consumer-focused perspectives provide substantive and testable hypotheses of recurrence. We think a producers-focused perspective is needed to better explain recurrent cultural features.

Consider some recurrent themes of the religious traditions before or outside organised, doctrinal religions typical of state societies (Barlev, Mermelstein, & German, 2017; Barrett, 2004; Boyer, 2001, 2019). In small-scale communities, religious representations focus overwhelmingly on the prevention or palliation of misfortune, which people see as caused by spirits, gods, ancestors or witches (Boyer, 2019, 2022; Strathern, 2019). From the consumption standpoint, these notions are culturally transmitted to the extent that they fit evolved expectations of potential threat in human minds (Lienard & Boyer, 2006). Now looking at the production side, it is remarkable that in most societies there are specialists in the production of such representations (Singh, 2018; Winkelman, 1986), whose interests may explain the focus on threats and prevention. Precaution psychology is a specialised cognitive system (Woody & Szechtman, 2011). When precautionary information is deemed plausible, it is generally not put to the test, providing a niche for claiming expertise without delivering valid information, which may explain why individuals are motivated to engage in such activities. Only some people manage to convince others that they are qualified to interact with potential threats as regards gods and spirits – the frequent use of trance is a signal of such capacities (Singh, 2018). Winners in this game receive benefits in reputation and social support.

In the domain of artistic production, too, producers’ interests may account for some properties of cultural products, beyond what is explained by a consumption model. In most human societies, some people are strongly motivated to produce sculpture, music or painting. This creates, even in small-scale communities, a competition between individuals that leaves only some individuals as recognised artists with a valuable contribution, which requires special capacities but also, in most cases, long training and sustained effort. Against these costs, groups deliver reputational and material benefits to the recognised artists, which would explain the motivation to engage in such activity. This also explains why the products have to be designed to advertise those special qualities that make the artist uniquely qualified. For instance, visual arts are generally designed to signal agency, that is, the fact that they exist because of a creator's intentions (Gell, 1998). More precisely, painting and sculpture in most cultures must signal both (a) what they are about, what the representational intention was, and (b) that they are very difficult to produce, creating a ‘sweet spot’ with e.g. obviously skillful figurative representations as a preferred genre (Dutton, 2009). In music, too, these constraints are at work, which would explain why most successful musical genres remain close to intuitive tonal expectations (signalling to listeners that it is not random), while delivering anticipation and surprise (signalling the unique skills of the musician) (Huron, 2006; see also Huron, 2008; Lerdahl & Jackendoff, 1985).

Those are only sketches of potential hypotheses, aiming to address the proximate question, of the motivations and capacities engaged in the production of ‘symbolic’ culture. Considering the producers’ interests also requires that we address the ultimate question, of the selective pressures on such motivations and capacities. The present argument does not entail that we should postulate specific adaptations for the production of shamanism or tonal music or moral norms. However, it does suggest that we should consider what specific psychological adaptations are required, so that individuals may intuitively consider the potential fitness benefits of such activities. Only specific empirical research, in these various domains of culture, can address this question, as behaviour by itself is not transparent evidence for the psychological mechanisms that produce it (Tooby & Cosmides, 1992, 2005). Yet such research cannot even start, unless we recognise that there *are* potential fitness benefits to the behaviours that lead to cultural transmission.

## 5. Conclusion: cultural products as parts of an extended phenotype

Clearly, the domain of ‘symbolic’ culture is not a proper scientific domain – the term only denotes the apparent difficulty in explaining a great variety of cultural phenomena in terms of practical goals or direct fitness benefits. Indeed, we showed that there may be very different evolutionary dynamics at work in different situations.

Here we outlined three major pathways in the creation of symbolic culture – apart from the altruistic pathway, whereby individuals create, e.g. lullabies or shelters for the benefit of their kin: (a) in a manipulative (genetically selfish) pathway, producers utilise for their own benefit some consumers’ vulnerabilities, e.g. creating religious cults or manufacturing recreational drugs; (b) in a cooperative form of signalling, some producers may emit honest signals of their cognitive capacities, as a proxy for the genetic qualities under sexual selection; and (c) in the mutualistic pathway, consumers and producers jointly receive fitness advantages, e.g. in producing norms or increasing the probability of success of collective action.

These models naturally extend to modern conditions, in which most cultural production and consumption in large-scale societies consists of contract-based market transactions for songs, paintings, novels, etc. Market conditions of course result in distinct trends of cultural evolution, very different from those observed where direct physical co-presence is required (Morin, 2016). In particular, the amplitude and speed of diffusion are dramatically different from those observed in small-scale communities (Acerbi, 2016). However, the logic of fitness advantages remains the same.

Only empirical evidence can adjudicate which of these pathways is more appropriate as a model for a particular situation of cultural production and consumption. Yet in all domains, the focus on producers’ as well as consumers’ fitness benefits helps us understand what cognitive capacities are under selection, which in turn suggests new hypotheses to explain the particular features of successful cultural products.

We argued that cultural products are not or not just *by-products* of the human mind's architecture but should also be considered as direct *products* of other minds, which like any other behaviour should be considered in terms of fitness.

This perspective amounts to describing cultural production as part of the human extended phenotype (Borau & Bonnefon, 2020; Sterelny, 2018). The term denotes the fact that a phenotype should not be limited to the result of direct gene expression (protein biosynthesis or tissue growth), but should extend to all resulting manifestations of gene expression inside or outside the individual organism. The best-known examples are some animals’ capacity to modify their environment using architectural constructions (spider's web, beaver's dam, termite mound). In the same way, producing convincing shamanistic cures or telling good stories may increase the fitness of the shaman or the story teller. That is why the cognitive faculties (theory of mind, language) involved in belief creation or joke telling, and in all other domains of cultural creation, are under selection. This gene-centred view of cultural production may generate new hypotheses to explain the features of successful cultural products. The extended phenotype, in our model, resides in the spread of particular mental representations in other minds.

This approach turns upside down our intuitive notion of culture. Culture is not ‘something’ that acts on humans, rather individuals have an impact on others through their cultural products. Culture is not ‘transmitted’ but humans use other people's productions (beliefs, tools, buildings) to further their own goals, which sometimes (but not always) leads to transmission and (rarely) results in cumulative processes.

## Data Availability

No original data are reported in this article.
